# Droplet Microfluidics for the ex Vivo Expansion of Human Primary Multiple Myeloma Cells

**DOI:** 10.3390/mi11030261

**Published:** 2020-02-29

**Authors:** Pilar Carreras, Iciar Gonzalez, Miguel Gallardo, Alejandra Ortiz-Ruiz, Joaquin Martinez-Lopez

**Affiliations:** 1 CSIC, Spanish National Research Council, 28006 Madrid, Spain; iciar.gonzalez@csic.es; 2 Hospital 12 Octubre, Hematology Department, Research institute i+12, 28040 Madrid, Spain; mgallardo@cnio.es (M.G.); mortizru86@gmail.com (A.O.-R.); jmartinezlo1967@gmail.com (J.M.-L.); 3 CNIO, Spanish national cancer research Centre, Hematological malignancies research unit, 28029 Madrid, Spain; 4 UCM, Complutense University Madrid, Medical faculty, 28040 Madrid, Spain

**Keywords:** microdroplets, microfluidics, stem cell encapsulation

## Abstract

We previously reported a new approach for micromanipulation and encapsulation of human stem cells using a droplet-based microfluidic device We demonstrated the possibility of encapsulating and culturing difficult-to-preserve primary human hematopoietic stem cells using an engineered double layered bead composed by an inner layer of alginate and an outer layer of puramatrix constructed using a soft technology without the use of any external force. In this work, we use this micro manipulation technique to build a 3D scaffold as a biomimetic model to recapitulate the niche of patient-derived multiple myeloma cells (MM cell) using a multilayered 3D tissue scaffold constructed in a microfluidic device and cultured in 10% FBS culture medium. In the current study, we included the use of this biomimetic model comprising supporting human Mesenchymal stem cells to show the mid-term survival of MM cells in the proposed structures. We found that the generated microniches were suitable for the maintenance of MM cells with and without supporting cells. Additionally, cultured MM cells in droplets were exposed to both Bortezomib and Lenalidomide to test their toxicity in the cultured patient derived cells. Results indicate that the maintained MM cells were consistently responding to the applied medication, opening a wide field of possibilities to use the presented micro device as an ex vivo platform for drug screening.

## 1. Introduction

Previous studies have been reported for the recapitulation of the stem cell microniche by droplet microfluidics [1]. Ex vivo maintenance and expansion of primary human multiple myeloma (MM) cells have presented several difficulties [2] due to the lack of an in vitro method able to reproduce the complex bone marrow niche microenvironment.

Some tissue models have been reported for the ex vivo culture of primary MM cells [3,4,5]. Whereas these approaches have been successful in achieving MM cells maintenance for a time span of several weeks, the process to reach these results has been quite complex as several supporting cells and/or complex perfusion systems were requested [6]. Microfluidics have recently arisen as a promising technology for 3D cell culture as it provides the possibility of generating biomimetic structures in which native stem cell biological processes can be recapitulated [7]. Three-dimensional cell culture systems have included many scaffold-free [8,9] and scaffold supported systems [10,11]. Stem cell maintenance studies in these structures have also been performed in suspension or in a spinner flask, resulting in a large heterogeneity in spheroid size [12], and in hanging-drop techniques that provide some control over the spheroid size but which lack aggregated generation control [13,14].

Whereas several methods able to form an aggregation of cell structures have been reported, most of them showed either a lack of stability on the template [15], or control on the geometry of the cell constructs [16]. Previous studies have reported the generation of a MM niche by microfluidics techniques [17]. However, these techniques were complex to perform and most of the time soluble additive factors were required for its growth and maintenance [18,19].

Hydrogels have been proved to be successfully used for 3D cell culture purposes [20,21]. While alginate is the most well-known hydrogel in cell culture studies, there is a growing interest in microengineering materials for improving the extracellular matrix recapitulation [22].

Recently we reported a method in which a double layered hydrogel bead able to accommodate adult stem cells where controlled generation of the structure geometry, composition and cell distribution was achieved [1]. This technology has been improved and accommodated to culture MM stem cells as part of the wide field of applicability that the method can offer for life science studies.

In the presented droplet-based microdevice, hydrogel droplets are produced by hydrodynamic focusing techniques and gelled by the circulation of the droplet between two laminar flows, one containing the cross-linking agent. This generated droplet is coated by another synchronized hydrogel bead as previously reported, generating on demand three-dimensional niches for stem cell allocation by synchronizing droplet production rates. The combination of these processes of droplet generation and passive mixing without the use of external forces makes this technology suitable for the encapsulation of stem cells [1].

The presented method permits the rapid extraction of the generated cellular constructs for immediate washing and culture, limiting the time exposure of the cells to the cross-linking agent. This feature allows the successful culture of cells like MM cells which usually present difficulties to be maintained ex vivo. This new technology does not require a complex setup or affective growth factor and represents a promising technique able to provide optimal requirements for 3D multiple myeloma (MM) stem cell culture for personalized medicine applications and ex vivo drug testing.

## 2. Materials and Methods

### 2.1. Materials/Chemicals

Mineral oil (Sigma Aldrich, St. Louis, MO, USA) was used as the main oil carrier. Sudan red dye (Sigma Aldrich) was used to color the oil containing the cross-linking agent and visualize the interface between the two main carrier oil flows. Alginic acid sodium salt powder (Sigma Aldrich) was used to prepare the alginate solutions. The aqueous phase was composed of deionized water unless otherwise stated. Puramatrix 0, 3% (Becton Dickinson) solution was prepared at the corresponding concentrations by sonication of the gel for 30 min at 30 °C. Acetic acid (Sigma Aldrich) was used as the cross-linking agent. Nano-calcium carbonate powder was purchased from Skyspring Nanomaterials (Houston, TX, USA) and used following the vendor instructions (https://www.ssnano.com).

### 2.2. Cell Assays

Phosphate Buffered Saline PBS was acquired from Cultek. Calcein-AM (0.1 ug/uL) and DAPI (4′, 6-diamidino-2-phenylindole), 10 mg (200ug/mL) were purchased from Sigma-Aldrich (St. Louis, MO, USA). CD38 FITC (fluorescein isothiocyanate) was purchased from Becton Dickinson, US. Phosphate Buffered Saline PBS was acquired from Cultek (Madrid, Spain).

Patient derived MM cells were collected and inmunomagnetically purified with written consent in accordance with the Institutional Review Board of Hospital 12 October Madrid and in accordance with the Declaration of Helsinki.

Patient: MM IgG Kappa IIIA ISS I patient treated with VTDx6 + TASPE regimen, Rt +KRD after 1st relapse, PomCyDex + RT after 2nd relapse and Selinexor+Bortezomib+Dexametasone regiment after 3rd relapse, in partial response at the moment of the sample.

Human Mesenchymal stem cells were purchased from DSMZ. The cell line was cultured in DMEM (Biowest, France) supplemented with 10% fetal bovine serum. Cells were cultured and used from passages 2–6 in a 37 °C humidified incubator with 5% CO2. Trypsin-EDTA (0.25%), phenol red was acquired from Thermo Fisher Scientific (Waltham, MA, USA).

### 2.3. Microfabrication

The microfluidic devices were designed using Solidworks CAD (Dassault Systèmes, Vélizy-Villacoublay, France) software. A computer numerical control (CNC) program was then created using Mastercam (CNC Software Inc., Tolland, CT, USA), according to the microfluidic circuit design and dimensions. The outline of the device was extracted from a PMMA (polymethylmethacrylate) plastic sheet (MacMaster-Carr, Elmhurst, IL, USA) using a Laser engraver (VLS 6.30, Universal Laser Systems, Scottsdale, AZ, USA). The plastic cut out was then taken to a CNC machine (HAAS Super Minimill, Oxnard, CA, USA), where the microfluidic circuit was created based on the Mastercam program. Milling conditions: milling direction, spindle speed and feed rate were optimized for surface quality. Finally, fluidic ports were created using a drill press (Ellis).

**Figure 1:**
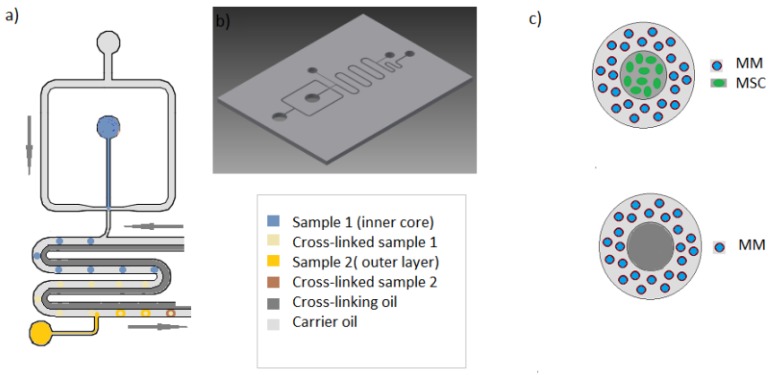
(**a**) Schematic of the double layered hydrogel bead generator. (**b**) Autocad layout of the microfluidic device. (**c**) MM (multiple myeloma) microniche models used. Top: double layered bead containing human multiple myeloma cells in the outer layer and human Mesenchymal stem cells in the inner core. Bottom: double layered bead containing human MM cells in the outer layer and an empty alginate core.

The PMMA device was sealed with a self-adhesive pouch (Opko diagnosis, Miami, FL, USA). Main channel dimensions are displayed in Figure 1 The device was connected to open barrels of 10 mL disposable plastic syringes (BD biosciences) via silicone tubing (ID 1.1 mm O.D. 2.16 mm) (Fisher scientific). The driving force used for the experiments was gravity, so liquid pressure on the microdevice was controlled by the heights of the open syringes. Pieces of polytetrafluoroethylene (PTFE) tubing (OD 2 mm) (Cole Parmer, Vernon Hills, IL, USA) were used to interface the reservoirs to the chip.

### 2.4. Microscopy

Fluorescence images were acquired using inverted laser scanning confocal microscopy (LSM510 META, Axiovert RT 200M, Zeiss, Oberkochen, Germany) equipped with an environmental chamber maintaining the cells under physiological conditions, using the laser lines 405 nm and 488 nm. For all other experiments, a Motic Stereoscope (Motic, Hong Kong, China) and Moticam 2.0 software (Motic) were used.

### 2.5. Research Methodology

The schematic of the device used to encapsulate MM cells is depicted in Figure 1.

First, alginate droplets containing calcium carbonate nanopowder are generated by hydrodynamic focusing using gravity to drive pressure. The droplets then travel through a meandering channel containing two laminar oil flows. By the addition of acetic acid to the second laminar oil flow continuous phase, calcium ions are released after droplet formation by the diffusion of the acetic acid across the oil interface. The so generated droplets are then passed by a second inlet were a stream of second sample layer alginate droplets are generated in a synchronous way.

Hence, when the inner core droplet reaches the area of the second droplet production, the two droplets (one gelated and another ungelated) passively mix along the meander, generating a double layered hydrogel bead. This process was described in detail in the previously reported work [1].

**Figure 2:**
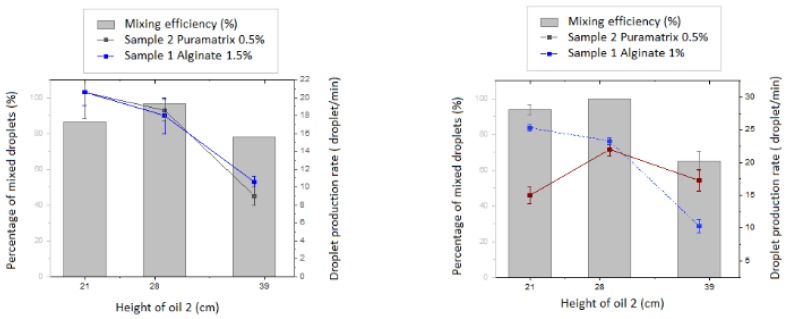
Characterization for the double layered device operational conditions using droplet different combinations of Puramatrix(0.3%) and alginate left: (1.5%) and right:(1%) containing calcium nano powder (1 wt%).

Further characterization of the mixing efficiency of the reported device is depicted in Figure 2.

## 3. Results

Samples were generated by premixing aqueous samples containing alginate (1.5 wt%) and Calcium carbonate nanopowder (1 wt%) and were prepared as sample 1 for the generation of the inner core. Puramatrix (0.3% v/v) was prepared following the vendor instructions for the generation of the outer layer. The second oil was prepared by adding glacial acetic acid to a pre-dyed Sudan red mineral oil (0.5% v/v).

Adjustments of the samples’ flow rates were performed so that the inner core produced was synchronized with the secondhand allowing its collection (Figure 2). Once the experimental flow rates were set up for the synchronization of the inner core droplet and outer layer droplet, characterization studies were run by varying the height of the oil 2 (oil containing the gelating agent) and using alginate as a scaffold in the inner core and puramatrix in two different concentrations for the outer layer. These measurements confirmed the already demonstrated capacity of the device to synchronize the hydrogel beads for the experimental conditions to study with the MM cells.

MM cells were purified from a specific patient. The suitability of the presented method for MM cell encapsulation was demonstrated by accommodating primary patient derived human Multiple Myeloma cells in the outer layer. Three different conditions were set in the same experiment. The first set of experiments included the generation of a mixed droplet containing only human MM cells. A second set of collected beads was planned so that the outer layer contained MM cells and the inner layer consisted of empty alginate. A third set of experiments was prepared so that human MM cells were located in the outer layer and supporting human Mesenchymal stem cells (hMSCs) were encapsulated into the middle layer, as these cells are naturally residing on the MM microniche.

Prior to resuspension in PBS and mixture in premixed alginate emulsification, human Mesenchymal stem cells were routinely cultured and trypsinized. We used 5 × 106 cells/mL for human MM and 0.7 × 106 cells/mL for hMSC.

The generated cell laden structures were collected and washed to remove residual acid. FITC CD38 which fluoresces green upon the reaction of intracellular esterase and stains live cells, and DAPI 0.1 M which binds to the DNA of dead membrane compromised cells were employed to visualize the distribution of human MM alive and dead cells right after encapsulation.

**Figure 3:**
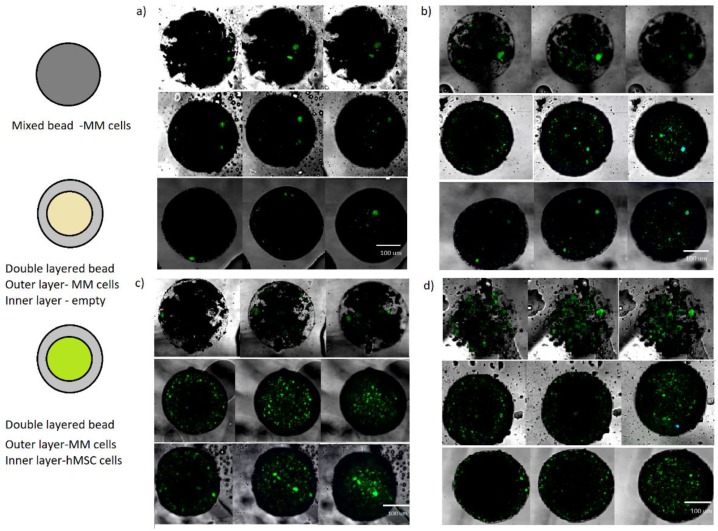
Collected beads after (**a**) 24 h, (**b**) 48 h, (**c**) 72 h, and (**d**) one week. For each case from top to bottom: mixed bead with MM cells, double layer bead with an outer layer of MM cells and empty inner alginate core and double layer bead with an outer layer of MM cells and inner layer of human Mesenchymal stem cells (hMSC) cells. For each case from left to right: different Z plane cuts selected to include the maximum number of positive cells.

Figure 3 shows the results of the collecting of these three sets of beads after 24, 48, 72 h and one week of culture in 10% FBS (Fetal Bovine Serum) DMEM. Collected beads were imaged under confocal microscope; images were taken at the middle plane of the bead and then three more planes to the bottom along the z axis in order to visualize the maximum number of encapsulated cells.

**Figure 4:**
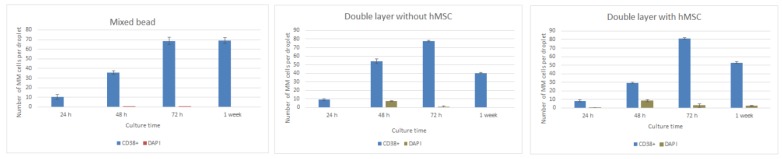
Number of FITC CD38+ and DAPI cells for the studied cases. From left to right: counted cells for mixed beads, double layered beads without MSCs and Double layered beads with MSCs.

Images were analyzed for the first week of culture and cells were manually counted using Image J software. Cells were manually counted over a minimum of ten beads per condition and taking into account cells imaged at least in four planes along the z axis in order to quantify the number of positive FITC CD38 cells and DAPI. It was observed that cell numbers were increasing significantly during the first 72 h. After a week cell numbers were slightly decreased for the double layered beads and maintained for the mixed bead case. Results are compiled in Figure 4.

Beads were maintained in culture for two weeks performing three media changes per week. After two weeks of ex vivo MM culture, the collected beads were incubated with Bortezomib and Lenalidomide for 24 h.

The frontline therapy for multiple myeloma patients is the combination of three drugs, bortezomib, lenalidomide and dexametasone. Bortezomib is a proteasome inhibitor (binds to 26S catalytic subunit) with high efficacy against multiple myeloma plasma cells. The in vitro IC_50_ in MM cell lines varies in a range of 2–40 nM, that is the reason 8 nM and 16 nM concentrations were selected for the experiment.

On the other hand, lenalidome is an IMID (Immunomodulatory imide) drug. Lenalidome has a plethora of different targets (e.g., CRBN) and effects (e.g., immunomodulation, anti-angiogenic, pro-apoptotic, anti-osteoclastogenic) and so it could be impacted in MM plasma and MM MSC cell. Due to its ample mechanism of action, it has a limited and lower effect in vitro compared with in vivo, with uM IC_50_ ranges (1–10 uM).

**Figure 5:**
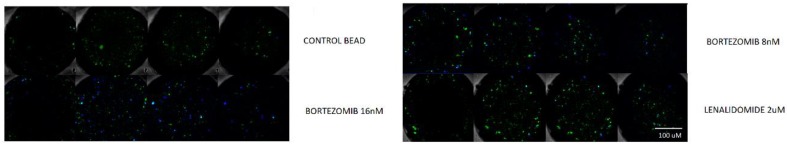
Confocal microscopy imaging of 4 different z planes for beads marked using FITC CD38 and DAPI. Z planes were chosen to image the maximum number of cells. From top to bottom: control bead, bead incubated with bortezomib 8 nM, bead incubated with lenalidomide 1 uM and bead incubated with lenalidomide 2 uM.

Therefore, the first test was designed to test bortezomib (concentration 8 nm) and lenalidomide (concentrations 1 uM and 2 uM (Figure 5)).

**Figure 6:**
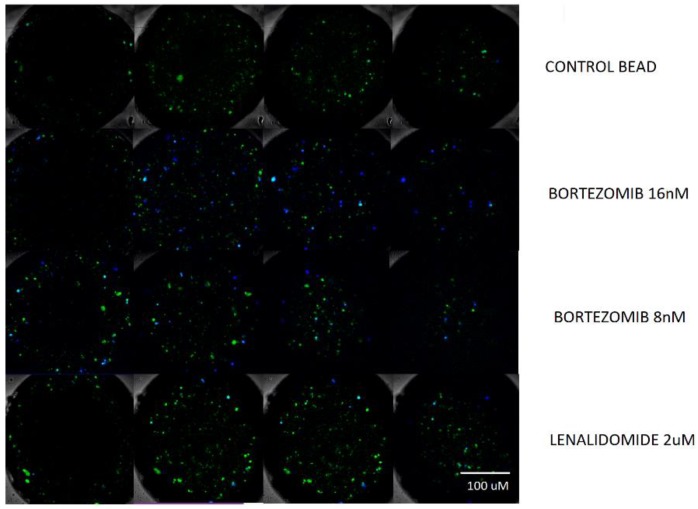
Confocal microscopy imaging of 4 different z planes for beads marked using FITC CD38 (green) and DAPI (blue). Z planes were chosen to image the maximum number of cells. From top to bottom: control bead, bead incubated with bortezomib 16 nM, bead incubated with bortezomib 8 nM and bead incubated with lenalidomide 2 uM.

It was observed that cells were responding significantly to the incubation process with bortezomib 8 nM and very slightly responding to the incubation with lenalidomide 1 uM and 2 uM. To assure this conclusion, experiments were repeated two days afterwards exposing the beads to a double concentration of bortezomib 16 nM, 8 nM and lenalidomide 2 uL. Frames extracted from the confocal imaging are displayed in Figure 6.

**Figure 7:**
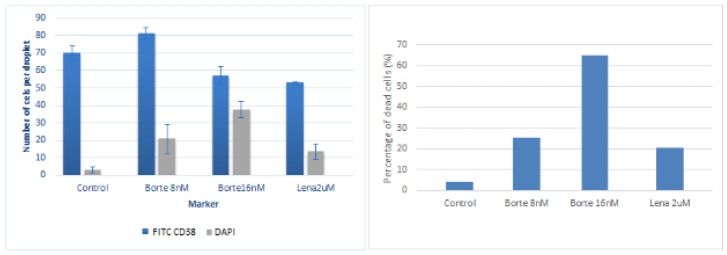
**Left**: Number of FITC CD38 and DAPI cells per bead for the studied cases (control, bortezomib 16 nM, 8 nM and lenalidomide 2 uM). **Right**: Percentage of dead cells for control beads, bortezomib 1 nM and 8 nM and lenalidomide 2 uM.

Cells were manually counted over a minimum of ten beads per condition and taking into account cells imaged at least in four planes along the z axis in order to quantify the number of positive FITC CD38 cells and DAPI. Results are shown in Figure 7.

Results, for these specific patient cells, suggest as expected that the patient is responsive to bortezomib significantly whereas the effect of lenalidomide is much lower. The results displayed for these specific patient cells in Figure 7 showed the correct efficacy of both anti-myeloma drugs against MM primary plasma cells (CD38+) in the generated microniche. Thus, this experiment is a proof of concept that the generated MM microniche is a novel vehicle allowing a successful culture of primary MM cells which are very difficult to culture in other conditions, such as 2D-culture or co-cultures. This could prove useful as a drug test platform.

These studies also indicate that the maintained MM cells were consistently responding to the applied medication, observing similar effects of anti-myeloma cells against MM cell lines in standard 2D-culture, opening a wide field of possibilities to use the presented micro device as an ex vivo platform for drug screening.

## 4. Discussion and Conclusions

The difficulties to culture primary plasma cells of multiple myeloma patients suggest the urgency to develop a novel method to study the MM PC (multiple myeloma plasma cells) ex vivo. In the current manuscript we describe a novel protocol to successfully harvest and amplify MM PC in mid/long-term cultures. The presented study indicates that MM cell viability is successfully maintained over at least two weeks with the generation of the presented microniches by microfluidic techniques. The beads at week 2 showed still high viability which suggests its longer-term applicability. Patient derived cultured cells in droplets were also exposed to the patient’s treatment obtaining corresponding responses. These results suggest that these ex vivo maintained MM cells are functional and could be used as a therapeutic platform.

The applications of this finding are remarkable. For instance, it could be used to amplify CD138+ cells in order to be applied for in vivo applications. However, the most prominent applicability probably is the use of therapeutic screening in this novel ex vivo platform with different regiment treatments (e.g., BTZ+Lenalidomide, secondary treatment lines, etc.) in order to determine to which treatment the cells are more sensitive

In conclusion, we present a novel droplet-based culture system for the encapsulation and maintenance of human primary multiple myeloma (MM) stem cells. This novel technology is based on a passive mixing principle and a gelation system in which the use of a double laminar oil flow where only one contains the cross-linking agent allows the mild generating of double layered hydrogel beads containing stem cells on demand. The soft consecutive coating of the inner core with a second layer without exposing the encapsulated cells to external forces that might reduce their viability constitutes the main technology advance towards a platform for 3D stem cell encapsulation.

Additionally, the suitability of the presented technology for encapsulation and maintenance of primary MM stem cells is demonstrated by using both only the studied MM stem cells and a combination of the MM stem cells and human Mesenchymal Stem cells (hMScs) in the inner layer as supporting cells. From the culture device perspective, our results demonstrate the possibility of replicating the multi-cellular MM niche in a simple and low-cost way and the possibility of a drug test directly over the cultured beads, characterizing the responses with confocal microscopy techniques.

The presented device represents an efficient attempt to engineer stem cell niches ex vivo using a three-dimensional matrix, and a novel platform to study MM cell behavior in vitro under controlled conditions as well as a novel therapeutic platform to study drug effects directly on patient derived cultures. Therefore, the presented device is a valuable and unique tool towards personalized drug testing and understanding stem cell behavior in 3-dimensional environments.

## References

[1] Carreras P., Chaves R.C., Gallardo M., Ortiz A., Martinez-Lopez J., Sia S.K. (2018). Microengineering double layer hydrogel structures towards the recapitulation of the hematopoietic stem cell niche. Sci. Bull..

[2] Kirshner J., Thulien K.J., Martin L.D., Marun C.D., Reiman T., Belch A.R., Pilarski L.M. (2008). A unique three-dimensional model for evaluating the impact of therapy on multiple myeloma. Blood.

[3] Calimeri T., Battista E., Conforti F., Neri P., Di Martino M.T., Rossi M., Foresta U., Piro E., Ferrara F., Amorosi A. (2011). A unique three-dimensional SCID-polymeric scaffold (SCID-synth-hu) model for in vivo expansion of human primary multiple myeloma cells. Leuk..

[4] Reagan M., Mishima Y., Glavey S.V., Zhang Y., Manier S., Lu Z.N., Memarzadeh M., Zhang Y., Sacco A., Aljawai Y. (2014). Investigating osteogenic differentiation in multiple myeloma using a novel 3D bone marrow niche model. Blood.

[5] Zhang W., Gu Y., Sun Q., Siegel D.S., Tolias P., Yang Z., Lee W.Y., Zilberberg J. (2015). Ex Vivo Maintenance of Primary Human Multiple Myeloma Cells through the Optimization of the Osteoblastic Niche. PLoS ONE.

[6] Dezorella N., Pevsner-Fischer M., Deutsch V., Kay S., Baron S., Stern R., Tavor S., Nagler A., Naparstek E., Zipori D. (2009). Mesenchymal stromal cells revert multiple myeloma cells to less differentiated phenotype by the combined activities of adhesive interactions and interleukin-6. Exp. Cell Res..

[7] Edmondson R., Broglie J.J., Adcock A.F., Yang L. (2014). Three-Dimensional Cell Culture Systems and Their Applications in Drug Discovery and Cell-Based Biosensors. ASSAY Drug Dev. Technol..

[8] Karadag A., Oyajobi B.O., Apperley J.F., Russell R.G., Croucher P.I. (2000). Human myeloma cells promote the production of interleukin 6 by primary human osteoblasts. Br. J. Haematol..

[9] Lin R.Z., Chang H.Y. (2008). Recent advances in three-dimensional multicellular spheroid culture for biomedical research. Biotechnol. J..

[10] Baraniak P.R., McDevitt T.C. (2011). Scaffold-free culture of mesenchymal stem cell spheroids in suspension preserves multilineage potential. Cell and Tissue Research.

[11] Chen A.A., Tsang V.L., Albrecht D.R., Bhatia S.N. (2006). 3-D Fabrication Technology for Tissue Engineering. BioMEMS and Biomedical Nanotechnology.

[12] Faulkner-Jones A., Greenhough S., A King J., Gardner J., Courtney A., Shu W. (2013). Development of a valve-based cell printer for the formation of human embryonic stem cell spheroid aggregates. Biofabrication.

[13] Kurosawa H. (2007). Methods for inducing embryoid body formation: In vitro differentiation system of embryonic stem cells. J. Biosci. Bioeng..

[14] Banerjee M., Bhonde R.R. (2006). Application of hanging drop technique for stem cell differentiation and cytotoxicity studies. Cytotechnology.

[15] Xu S., Nie Z., Seo M., Lewis P., Kumacheva E., Stone H.A., Garstecki P., Weibel D., Gitlin I., Whitesides G.M. (2005). Generation of Monodisperse Particles by Using Microfluidics: Control over Size, Shape, and Composition. Angew. Chem. Int. Ed..

[16] Markx G.H., Carney L., Littlefair M., Sebastian A., Buckle A.-M. (2008). Recreating the hematon: Microfabrication of artificial haematopoietic stem cell microniches in vitro using dielectrophoresis. Biomed. Microdevices.

[17] Lee E.J., Park S.J., Kang S.K., Kim G.-H., Kang H.-J., Lee S.-W., Jeon H.B., Kim H.-S. (2012). Spherical Bullet Formation via E-cadherin Promotes Therapeutic Potency of Mesenchymal Stem Cells Derived From Human Umbilical Cord Blood for Myocardial Infarction. Mol. Ther..

[18] Zhang W., Lee W., Siegel D.S., Tolias P., Zilberberg J. (2014). Patient-Specific 3D Microfluidic Tissue Model for Multiple Myeloma. Tissue Eng. Part C: Methods.

[19] Gunn W., Conley A., Deininger L., Olson S.D., Prockop D.J., Gregory C. (2006). A Crosstalk Between Myeloma Cells and Marrow Stromal Cells Stimulates Production of DKK1 and Interleukin-6: A Potential Role in the Development of Lytic Bone Disease and Tumor Progression in Multiple Myeloma. STEM CELLS.

[20] Andersen T., Auk-Emblem P., Dornish M. (2015). 3D Cell Culture in Alginate Hydrogels. Microarrays.

[21] Devolver R., Kong H.J. (2012). Hydrogels for in vivo-like three-dimensional cellular studies. Wiley Interdiscip. Rev. Syst. Biol. Med..

[22] Geckil H., Xu F., Zhang X., Moon S., Demirci U. (2010). Engineering hydrogels as extracellular matrix mimics. Nanomed..

